# Explainability agreement between dermatologists and five visual explanations techniques in deep neural networks for melanoma AI classification

**DOI:** 10.3389/fmed.2023.1241484

**Published:** 2023-08-31

**Authors:** Mara Giavina-Bianchi, William Gois Vitor, Victor Fornasiero de Paiva, Aline Lissa Okita, Raquel Machado Sousa, Birajara Machado

**Affiliations:** Department of Big Data, Hospital Israelita Albert Einstein, São Paulo, Brazil

**Keywords:** melanoma, Grad-CAM, Grad-CAM++, Eigen-CAM, Score-CAM, LIME, explainability

## Abstract

**Introduction:**

The use of deep convolutional neural networks for analyzing skin lesion images has shown promising results. The identification of skin cancer by faster and less expensive means can lead to an early diagnosis, saving lives and avoiding treatment costs. However, to implement this technology in a clinical context, it is important for specialists to understand why a certain model makes a prediction; it must be explainable. Explainability techniques can be used to highlight the patterns of interest for a prediction.

**Methods:**

Our goal was to test five different techniques: Grad-CAM, Grad-CAM++, Score-CAM, Eigen-CAM, and LIME, to analyze the agreement rate between features highlighted by the visual explanation maps to 3 important clinical criteria for melanoma classification: asymmetry, border irregularity, and color heterogeneity (ABC rule) in 100 melanoma images. Two dermatologists scored the visual maps and the clinical images using a semi-quantitative scale, and the results were compared. They also ranked their preferable techniques.

**Results:**

We found that the techniques had different agreement rates and acceptance. In the overall analysis, Grad-CAM showed the best total+partial agreement rate (93.6%), followed by LIME (89.8%), Grad-CAM++ (88.0%), Eigen-CAM (86.4%), and Score-CAM (84.6%). Dermatologists ranked their favorite options: Grad-CAM and Grad-CAM++, followed by Score-CAM, LIME, and Eigen-CAM.

**Discussion:**

Saliency maps are one of the few methods that can be used for visual explanations. The evaluation of explainability with humans is ideal to assess the understanding and applicability of these methods. Our results demonstrated that there is a significant agreement between clinical features used by dermatologists to diagnose melanomas and visual explanation techniques, especially Grad-Cam.

## 1. Introduction

Melanoma is a skin cancer that is more lethal than all the other skin cancers combined, even though it accounts for less than 5% of all cases (1). The global incidence of melanoma rose from 11.8 to 17.5/100,000 inhabitants from 2003–2006 to 2011–2014 (2, 3). In Australia, one of the countries with the highest incidence of this pathology in the world, the number of deaths from melanoma of the skin increased from 596 in 1982 to 1,405 in 2019 (4). In 2021, in the U.S.A., 106,110 cases were diagnosed and 7,180 deaths by melanoma were estimated (5). Melanoma represents a high cost to society. Loss of productivity due to morbidity or premature death, as well as the cost of treatments, are a considerable burden for health systems and have multiple implications in the life of such individuals (6). It is ranked as one of the most expensive cancers, with a significant decrease in cost when diagnosed in the early stages (7, 8). The average cost per patient with melanoma ranges from € 149 for disease stage 0 to € 66,950 for stage IV (9). When melanoma is diagnosed early, it can be treated effectively and with a high probability of survival (5). Therefore, it is essential to promote prevention programs with periodic examination of the skin for the early detection of suspicious lesions to reduce the costs and mortality of melanoma (6). The ABCDE rule is a widely used method to recognize characteristics often associated with melanoma. It was developed for both physicians and patients. It includes: Asymmetry, Border irregularity, Color heterogeneity, Diameter larger than 6mm, and Evolution or transformation of the lesion over time (10).

Since the detection of melanomas at an early stage is essential for a good prognosis, and the distinction between melanomas and harmless pigmented lesions is often not trivial, AI-based classification systems may bring important contributions to this field. Artificial intelligence algorithms have performed *in silico* at least as well as expert dermatologists in detecting melanoma lesions (11–13). Results have been encouraging, but there are only a few recent studies trying to use AI in the real world to detect melanoma lesions (14–16). There is still some controversy about the use of AI for diagnoses in “real-life” clinical settings. Concerns include the possibility of biases, the lack of transparency and explainability, scalability, data integration and interoperability, reliability, safety, privacy, and the ethics of aggregated digital data (17, 18). As with any other innovation, especially in healthcare, AI must prove to be efficient, reliable, reproducible, and friendly enough to be accepted by those who are actually going to use it; in this case, physicians (or perhaps other health professionals) and patients. As for physicians, a recent study in Korea has shown that, in general, physicians have a positive attitude toward AI in medicine (19). Another study has presented similar results in a large international survey among dermatologists, indicating that AI is well-accepted in the dermatology field and that AI should be a part of medical training (20). As for patients, one article concluded that they expressed a high level of confidence in decision-making by AI and that AI can contribute to improving diagnostic accuracy, but should not replace the dermatologist (21). Another survey has shown that patients and physicians are willing to use AI in the detection of melanoma lesions. Patients appear to be receptive to the use of AI for skin cancer screening if implemented in a manner that preserves the integrity of the human physician-patient relationship (22).

To satisfy the requirement for transparent and comprehensible treatment decisions, it will be necessary to work on strategies that allow AI results to be interpreted and verified (at least in part). Due to the high complexity of the algorithms, complete transparency of AI will probably not be possible. Still, it may be possible to explain the decisive influencing factors on individual decision steps within the algorithms. Explainable artificial intelligence (XAI) is an initiative that aims to “produce more explainable models while maintaining a high level of learning performance (prediction accuracy); and enable human users to understand, appropriately trust, and effectively manage the emerging generation of artificially intelligent partners” (23). The aim of enabling explainability in ML, as stated by FAT (fairness, accountability, and transparency) (24), “is to ensure that algorithmic decisions, as well as any data driving those decisions, can be explained to end-users and other stakeholders in non-technical terms”.

For deep learning models, the challenge of ensuring explicability is due to the trade-off in terms of powerful results and predictions (25) and the inherent opacity of black box models. This represents a serious disadvantage, as it prevents a human being from being able to verify, interpret and understand the system's reasoning and how decisions are made (26). It is a common approach to understand the decisions of image classification systems by finding regions of an image that were particularly influential to the final classification. They are called sensitivity maps, saliency maps, or pixel attribution maps (27). These approaches use occlusion techniques or calculations with gradients to assign an “importance” value to individual pixels which are meant to reflect their influence on the final classification.

Gradient-weighted Class Activation Mapping (Grad-CAM) uses the gradients of any target concept flowing into the final convolutional layer to produce a coarse localization map highlighting important regions in the image for predicting the concept. It highlights pixels that the trained network deems relevant for the final classification (28). Grad-CAM computes the gradient of the class-score (called logit) with respect to the feature map of the final convolutional layer (28). Despite the difficulty of evaluating interpretability methods, some proposals have been made in this direction (29, 30). Grad-CAM is one method of local interpretability being used for deep learning models and was one of the few methods that passed the recommended sanity checks (29). There is also an improved version of the original Grad-CAM and CAM method, called Grad-CAM++. This method is based on the same principles as the original Grad-CAM method, but it uses a different weighted combination (31). Two other CAM techniques can be used: Eigen-CAM (32) and Score-CAM (33) which differ from the Grad-CAM by not relying on the backpropagation of gradients. A totally different approach can also be made using Local Interpretable Model-agnostic Explanations (LIME) technique, where the image is segmented into superpixels interconnected with similar colors (34).

To elucidate more about the explainability of deep neural network classification in melanoma lesions, we performed an exploratory experiment with 2 objectives. First, to assess the agreement rate between the features highlighted by 5 different techniques of visual saliency maps to the three most used clinical dermatological criteria for melanoma lesions: asymmetry, border irregularity, and color heterogeneity (ABC rule). Second, to subjectively evaluate the preferable techniques ranked by the dermatologists, the reasons for it and the degree of agreement between the two dermatologists about the five techniques.

## 2. Methodology

In this section, we will introduce the dataset used to build the classification model for evaluating the visual explanations, the Convolutional Neural Network (CNN) models used for the segmentation and classification tasks, the explainability methods used for the visual explanations, and the experiment performed. The development of the algorithm and its performance were described in detail in a previous article (35).

This study was approved by Hospital Israelita Albert Einstein Ethics Committees under the identification CAAE:32903120.40000.0071.1 and it is in accordance with the ethical standards on human experimentation and with the Declaration of Helsinki. Dermatologists that took part in the experiment signed consent forms agreeing to participate. This research was performed at Hospital Albert Einstein, São Paulo, Brazil, from January-March 2023.

### 2.1. Melanoma dataset

For this study, we used the following datasets: HAM10000 Dataset (36), MSK Dataset (37), Dataset BCN20000 (38), and Derm7pt (39), all publicly available. The first three datasets compose the dermoscopic image data available by ISIC (37–39), an international competition for the identification of skin diseases. Derm7pt is composed of clinical and dermoscopic images categorized by the 7-point technique for the identification of melanoma, with more than 2000 images of melanoma and non-melanoma. In this study, we selected only dermoscopic images. The total dataset consists of 26,342 images. Only two different classes were established for our dataset: melanoma (18%) and non-melanoma (82%).

### 2.2. Convolutional neural networks models (CNN)

The classification model for melanoma lesions was constructed using two steps: image segmentation and image classification. For the segmentation, we used the MaskR-CNN architecture (40). The lesions in the dermoscopy images were segmented and then used in the classification model in a way that the latter could focus only on the patterns closely related to the lesion itself, excluding most of the background information that could impair its classification capabilities. To train the segmentation model, we used 2000 images previously annotated by specialists with the regions of interest. Using transfer learning with a Resnet50 backbone and 20 epochs, the trained model reached a 99.69% mAP for our test set.

For the classification task, we divided the total dataset as 80% for training, 10% for validation, and 10% for testing the classification model. To train the model, we used the EfficientNetB6 convolutional neural network (41). This family of architectures achieved some of the best precision and efficiency in the literature (41), performing better than previous CNN (42, 43). Through transfer learning with pre-trained weights from the ImageNet (44), the model was fine-tuned for 50 epochs using the Adam optimization (45) with a 0.001 starting learning rate and a batch size equal to 32. The learning rate was scheduled to be reduced by a factor of 30% if the model failed to improve with a stagnant validation loss for 5 epochs. Finally, we used early stopping, also based on a validation loss of 10 epochs.

To address the imbalance in the two target classes, we trained the model using the focal loss function (46) to avoid bias for the most dominant class. We also weighted the classes according to their inverse frequency, in order to balance model attention in the loss function. All images were resized to 220 × 220. In addition, we applied data augmentation using common image processing operations (rotation, shear, horizontal flip, zoom). The sigmoid function was used to deliver the prediction result. In the tests, our model has achieved an average ACC of 0.81, AUC of 0.94, sensitivity of 0.93 and specificity of 0.79, considering the threshold of 0.5. More details of the model can be found in our study previously reported (35).

### 2.3. Explainability methods adopted

#### 2.3.1. Gradient-weighted class activation mapping (Grad-CAM)

Grad-CAM was proposed to produce visual explanations for decision-making in comprehensive classes of convolutional neural networks (28). The idea was to make AI models transparent and explainable, giving the possibility to identify flaws in the systems, mainly of deep learning models that were considered difficult to interpret. Some proposals have used Grad-CAM in an attempt to explain possible decisions of the model (47) in the medical field (48–51).

Since Grad-CAM does not require any particular CNN architecture, it can be used with fixed weights (after being trained), and it is able to explore the spatial information of the last convolutional layers through feature maps that are weighted and calculated, based on gradients. The positive values, which are the most “relevant” information for the classification result, can be obtained through a ReLU operation, defined as,


(1) $$\begin{array}{l} L_{Grad-CAM}^{c}=ReLU\left(\underset{k}{\sum }\alpha_{k}^{c}A^{k}\right) \end{array}$$

where $\alpha_{k}^{c}=\frac{1}{Z}\underset{i}{\sum }\underset{j}{\sum }\frac{\partial y^{c}}{\partial A_{ij}^{k}}$.

#### 2.3.2. Grad-CAM++

Grad-CAM++ technique is an improved version of the original Grad-CAM and CAM method. The Grad-CAM++ method is based on the same principles as the original Grad-CAM method, but it uses a weighted combination of the positive partial derivatives of the last convolutional layer feature maps with respect to a specific class score as weights to generate a visual explanation for the class label under consideration (Equation2) (31).


(2) $$\begin{array}{l} L_{Grad-CAM}^{c}=ReLU\left(\underset{k}{\sum }\alpha_{k}^{c}A^{k}\right) \end{array}$$

The class-discriminative saliency map generated by Grad-CAM++ is a high-resolution heatmap that indicates the regions of the input image that are most relevant to the specific prediction made by the network. For a given image, *Lc* is calculated as a linear combination of the forward activation maps, followed by a relu layer (Equation 3) (31).


(3) $$\begin{array}{l} L_{ij}^{c}=ReLU\left(\underset{k}{\sum }w_{k}^{c}A_{ij}^{k}\right) \end{array}$$

#### 2.3.3. Eigen-CAM

The Eigen-CAM technique leverages the principal components on the activation maps of the convolutional layers (32). It does not rely on the backpropagation of gradients. For the last convolutional layer:

1. Singular value decomposition (SVD) is used to factorize the combined activation map A for input *X* as *A* = *U*∑*V*^*t*^;
2. The activation map is then projected on the first eigenvector of the *V* matrix;
3. The projection highlights the principal components of the activation map.

In this method, there is no use of a ReLU activation function. Conceptually, the Eigen-CAM can be defined as,


(4) $$\begin{array}{l} L_{Eigen-CAM}=AV_{1} \end{array}$$

where *V*_1_ denotes the first the eigenvector at the first position in the *V* matrix.

#### 2.3.4. Score-CAM

Like Eigen-CAM, Score-CAM does not rely on the backpropagation of gradients. It borrows from the Grad-CAM technique in the sense that it is also non-dependent on a particular architecture; where they differentiate, however, is in the way they deal with the flow of gradient information. Instead of using the gradient from the last convolutional layer to build on the importance of each region of input *X* toward class *C*, the Score-CAM technique assimilates the importance of each region as an increase of confidence in the overall prediction (33). For a specific convolutional layer:

1. Each activation map is upsampled, normalized, and then used as a mask for input *X*, highlighting the most activated regions;
2. The masked input image is passed through the CNN resulting in a logit for each class;
3. All logits and activation maps are linearly combined;
4. A *ReLU* activation function is applied to the combined product, resulting in the Score-CAM output.

Because gradients can be noisy, explode, and/or vanish (52), these characteristics can also be present in the layer activations (53), thus resulting in suboptimal CAM visualizations. The Score-CAM technique, however, is not dependent on the model gradient.

Conceptually, the Score-CAM can be defined as,


(5) $$\begin{array}{l} L_{Score-CAM}^{k}=ReLU\left(\underset{k}{\sum }\alpha_{k}^{c}A_{l}^{k}\right) \end{array}$$

where $\alpha_{c}^{k}=C\left(A_{l}^{k}\right)$, and $C\left(A_{l}^{k}\right)=f\left(X\cdot H_{l}^{k}\right)-f\left(X_{b}\right)$.

#### 2.3.5. Local interpretable model-agnostic explanations (LIME)

LIME is model agnostic, which allows it to be utilized across a wide range of machine learning models. The locally weighted square loss (L) as the metric choice by authors (Equation 6). This loss function takes into account the exponential kernel *rx*(*z*), which is defined as exp(−*D*(*x, z*)^2^/σ^2^), where *D* represents a distance function, such as the cosine distance for text or the *L*2 distance for images, and σ is the width of the kernel (54).


(6) $$ℒ(f,g,\pi_{x})=\sum_{z,z^{\prime }\epsilon Z}\pi_{x}\left(z\right){\left(f\left(z\right)-g\left(z^{\prime }\right)\right)}^{2}$$

How LIME is used for image:

1. The image is segmented into superpixels. Superpixels are interconnected pixels with similar colors;
2. The surrogate model highlights the superpixels of the image that are the most active in predicting a certain class;
3. The image is transformed into a binary vector where 1 indicates the original superpixel and 0 indicates a grayed-out super-pixel.

The complexity depends on the time required to compute the prediction of the relevant class and the number of samples N. Due to this complexity, LIME may take longer than other methods, especially when applied to image data (34, 54). In the present publication, the LIME is used to highlight superpixels that have the maximum positive and negative influence on the model's prediction.

### 2.4. The experiment

In order to analyze the impact of the five different explainability techniques on humans, we defined two major questions to be addressed experimentally. They are:

1. Is there a quantitative agreement between dermatologists ABC rule and the visual explanation techniques for melanoma?
2. Do dermatologists qualitatively agree with the visual explanation techniques for melanoma?

In the next sections, we will explore each question in further detail.

#### 2.4.1. Is there a quantitative agreement between dermatologists ABC rule and the visual explanation techniques for melanoma?

In this experiment, we aimed to apply an explainability method visual analysis by human experts, such as dermatologists, comparing the highlighted areas in the saliency maps with the areas of the lesion that show asymmetry, border irregularity, and color heterogeneity (ABC rule), three of the main features evaluated in a melanoma lesion.

From the dataset, we selected 100 lesions correctly classified by the model as melanoma. These 100 dermoscopy images were analyzed by two experienced and Board-Certified dermatologists (MGB and ALO). They first assessed only the dermoscopy image and graded three of the five most frequently melanoma criteria (ABCDE) used in clinical practice: asymmetry (A), border irregularity (B), and color heterogeneity (C). They did not grade diameter (D) because most of the dermoscopy images had no scale measure and evolution in time (E) due to the fact that the clinical photographs in the dataset were taken at one point in time and no follow-up images were available.

**Figure 1:**
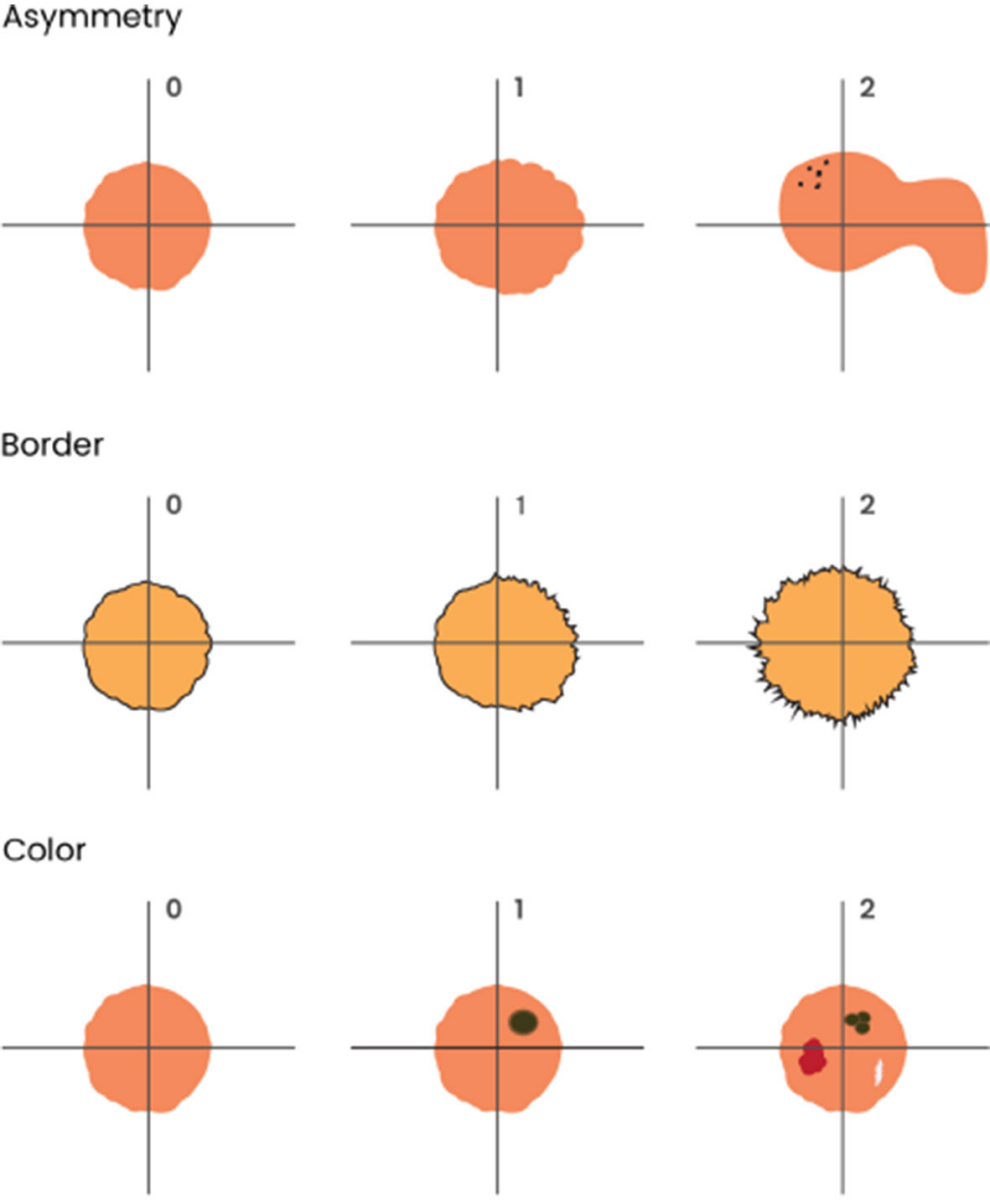
Graphical representation of ABC melanoma criteria used in clinical images: asymmetry, border irregularity, and color heterogeneity. To assess asymmetry, the lesion was divided into 4 quadrants, and its shape and color distribution were analyzed. If all 4 quadrants had regular shapes and colors, there was no asymmetry (0); if 2 or 3 quadrants were similar, there was mild asymmetry (1); and if all four quadrants were different, there was severe asymmetry (2). For borders, they evaluated shape and regularity. If the aspect was smooth and regular in color, the borders were considered benign (0); if ≤ 50% of the border area presented irregular borders or signs of color abnormality, it was considered as partial involvement (1), and if >50%, severe involvement (2). Finally, if >50% of the lesion's limits could not be evaluated, it was considered non-available (N/A). For color, we assessed the degree of color heterogeneity by the number of colors present in the lesion: presence of one color was considered as no heterogeneity (0); presence of two colors was considered as mild heterogeneity (1); presence of three or more colors was considered as severe heterogeneity (2).

Both dermatologists had to reach a consensus to use a semi-quantitative scale from 0 to 2 to grade the ABC features in the lesions, as shown in Figure 1. To assess asymmetry, the lesion was divided into 4 quadrants, and its shape and color distribution was analyzed. If all 4 quadrants had regular shapes and colors, there was no asymmetry (0); if 2 or 3 quadrants were similar, there was mild asymmetry (1); and if all four quadrants were different, there was severe asymmetry (2). For borders, they evaluated the shape and regularity. If the aspect was smooth and regular in color, the borders were considered benign (0). If ≤ 50% of the border area presented irregular borders or signs of color abnormality, it was called partial involvement (1), and if >50%, severe involvement (2). If >50% of the lesion's limits could not be evaluated, they were designed as non-available (N/A). For color, we assessed the degree of color heterogeneity by the number of colors present in the lesion: one color present, no heterogeneity (0); two colors present, mild heterogeneity (1); three or more colors present, severe heterogeneity (2).

**Figure 2:**
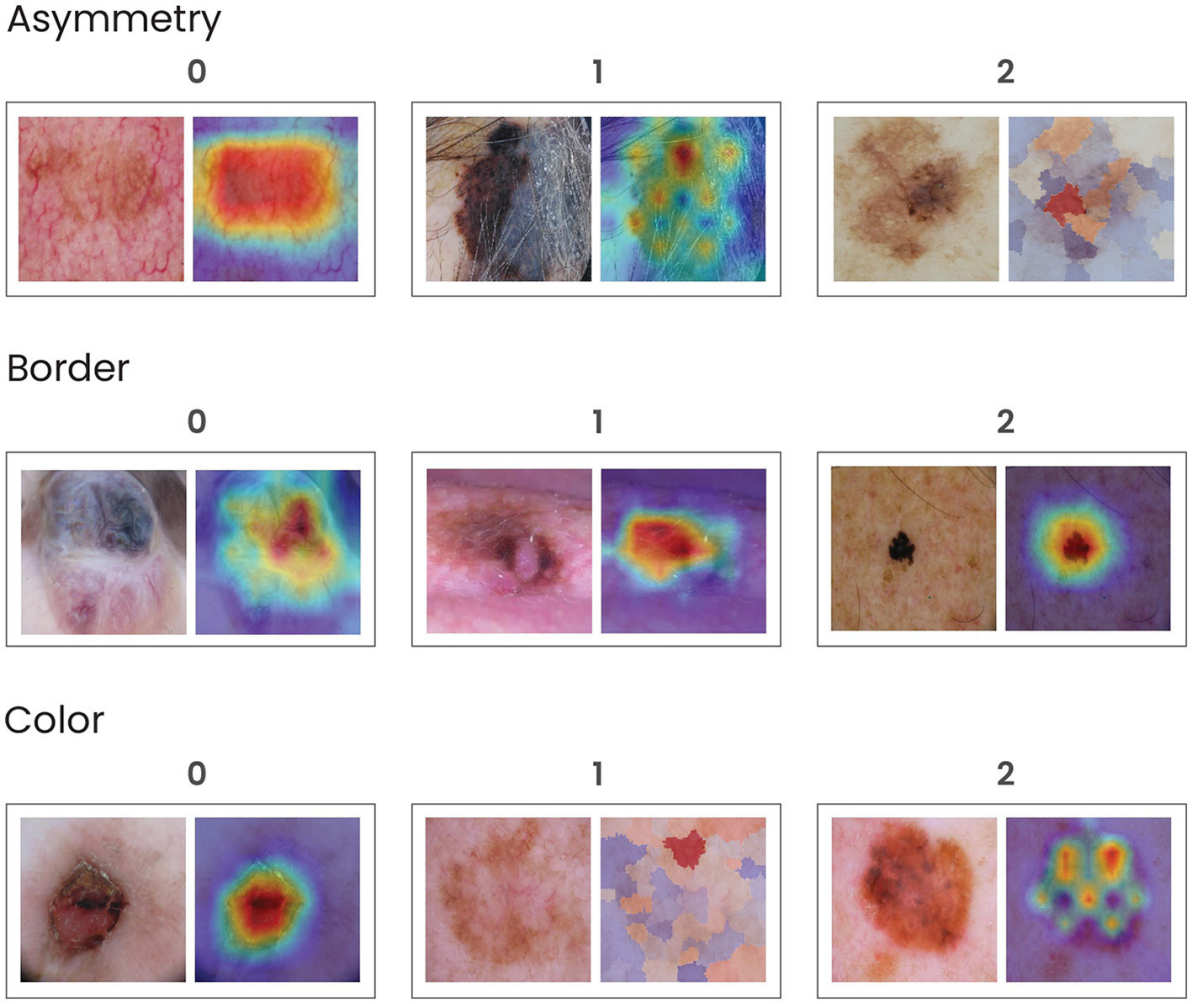
Grading examples of the visual map explanation techniques. For asymmetry, the visual explanation map was divided into 4 quadrants and shape and color distribution were analyzed. If all four quadrants showed the same color and format, there was no asymmetry (0); if 2 or 3 quadrants were similar, there was mild asymmetry (1); and if all four quadrants were different, there was severe asymmetry (2). For borders, the clinical border area was compared to the highlighted visual map. If the visual technique showed no highlight or ≤ 50% of the border area highlighted with cold colors, it was considered as no highlight (0). If ≤ 50% of the area was highlighted with warm colors or >50% with cold colors, it was considered partial border highlight (1); if >50% of the areas was highlighted with warm colors, it was considered total border highlight (2). Finally, if >50% of the lesion's limits could not be evaluated clinically, it was considered non-available (N/A). For color abnormality, dermatologists decided to compare the most significant color abnormalities in the dermatoscopy image as if they had a saliency map in their minds, comparing the imaginary heatmaps to the ones in the visual techniques. If the clinical color abnormalities presented an agreement area of ≤ 75% for warm colors, it was considered total agreement (0); if it was 25−75% for warm colors or >75% for cold colors, it was considered as partial agreement (1); if it was < 25% for warm colors or 25−75% for cold colors, it was considered total disagreement (2). For grading the highlight colors, we established blue/purple as cold colors and orange/red as warm colors.

Next, they analyzed each visual explanation technique (Grad-CAM, Grad-CAM ++, Eigen-CAM, Score-CAM, and LIME) in conjunction with its dermoscopy image, separately, in pairs, and blindly to the techniques name. For each of them, they assessed the features highlighted by the saliency map, using the following criteria (Figure 2). For asymmetry, it was the same criteria as for clinical features. The visual explanation map was divided into 4 quadrants and shape and color distribution were analyzed. If all 4 quadrants showed the same color and format, there is no asymmetry (0); if 2 or 3 quadrants are similar, there was mild asymmetry (1); and if all four quadrants were different, there is severe asymmetry (2). The clinical border area was compared to the highlighted visual map for borders. If the visual technique showed no highlight or ≤ 50% of the border area highlighted with cold colors for the clinical borders, it was classified as no highlight (0). If ≤ 50% of the area was highlighted with heat colors or >50% with cold colors, it was called partial border highlight (1). If >50% of the area were highlighted with heat colors, it was designated as total border highlight (2) or non-available (N/A), and if >50% of lesion's limits could not be evaluated clinically.

**Figure 3:**
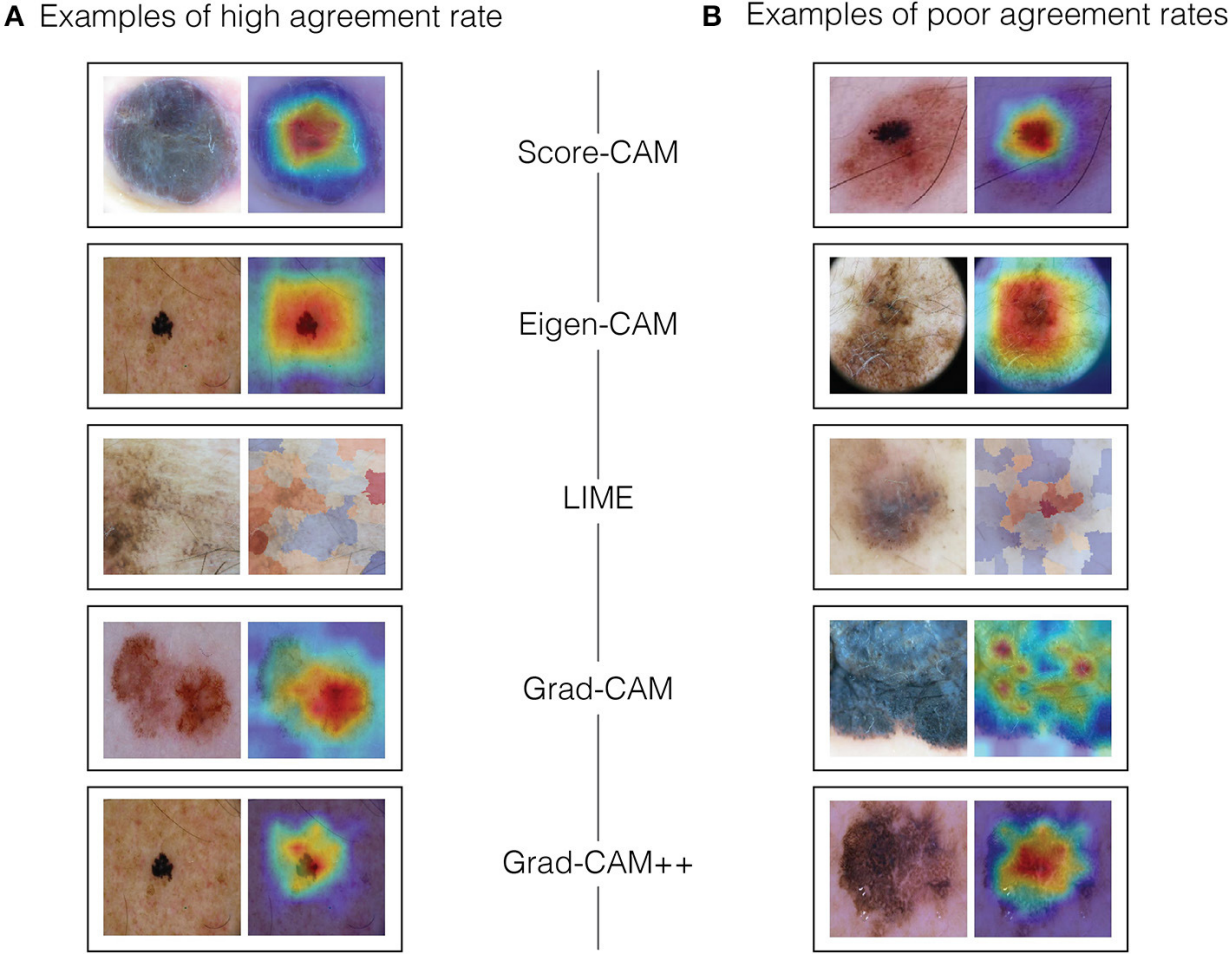
Examples of high and low agreement cases. **(A)** Examples of high agreement rate. **(B)** Examples of poor agreement rates.

For color assessment, we had to pursue a different strategy, mainly because visual heat maps, by definition, ought to display multiple colors, leaving all the maps to be rated as showing severe heterogeneity of colors (2), which would not be meaningful to the dermatologists understanding. Thus, dermatologists decided to compare the most significant color abnormalities presented in the dermoscopy image (as if they had a saliency map in their minds) to the heat colors of the visual map, considering its location and intensity, and grading the match between them. If the clinical color abnormalities presented an agreement area was ≤ 75% for heat colors, it was called total agreement (0). If the matched area was 25-75% for heat colors or >75% for cold colors, it was designated as partial agreement (1). If the matched area for heat colors was < 25% or 25-75% for cold colors, it was considered total disagreement (2). For grading the highlight colors, we established blue/purple as cold colors and orange/red for heat colors. Examples of high and low agreement cases can be seen in Figure 3.

To calculate the agreement rate between the clinical criteria and visual techniques, we used the following criteria: if the difference between their grade scales was zero, they were in total agreement. If the difference was one, they had a partial agreement and if the difference was two, they had no agreement. For example, if dermatologists graded the heterogeneity of colors as 0 in the clinical image and as 0 in the visual technique, the difference was zero, so they were in total agreement. On the other hand, if dermatologists graded border irregularity as 2 for the clinical image and as 0 for the visual explanation technique, the difference was 2, and therefore there was no agreement. At last, if the asymmetry was rated as 0 for the clinical image and as 1 for the explanation technique, the difference was 1, so that corresponded to a partial agreement.

#### 2.4.2. Do dermatologists qualitatively agree with the visual explanation techniques for melanoma?

**Figure 4:**
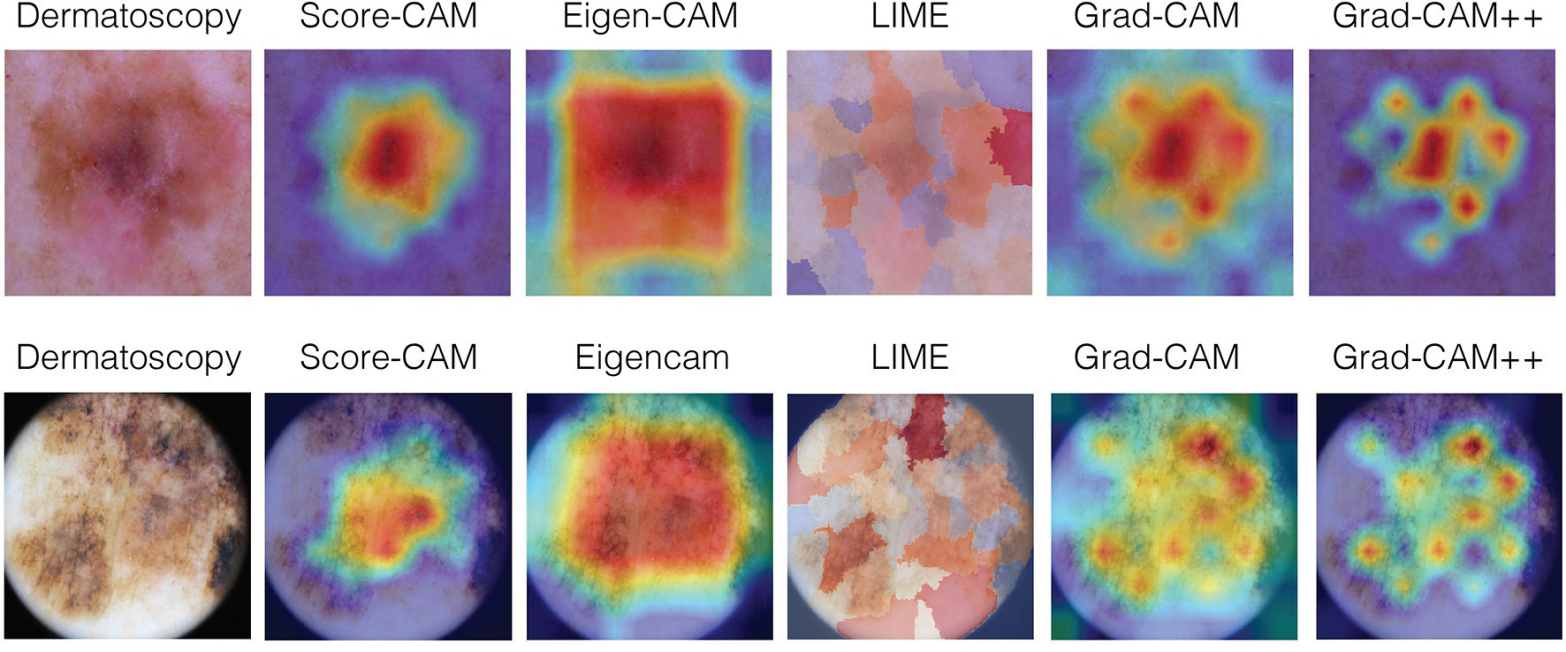
Examples of clinical melanoma images and their respective visual maps using Score-CAM, Eigen-CAM, LIME, Grad-CAM, and Grad-CAM++.

The rationale for this part of the qualitative study was to capture the overall characteristics perceived by the experts about each explainability technique, making comments about each of them and ranking their preferable techniques. For this purpose, after grading ABC, we showed all the images again, with the respective label for each technique to both dermatologists and asked them to make comments about each technique and how they would rank the techniques in order of the most preferable to the least (1-5). After that, they were also asked to read the comments and determine if they agree or not with the other experts observations, according to the following criteria: total agreement; partial agreement; no agreement nor disagreement; partial disagreement; and total disagreement. Examples of clinical melanoma images and their respective visual maps using Score-CAM, Eigen-CAM, LIME, Grad-CAM, and Grad-CAM ++ can be seen in Figure 4.

## 3. Results

### 3.1. Quantitative results

**Figure 5:**
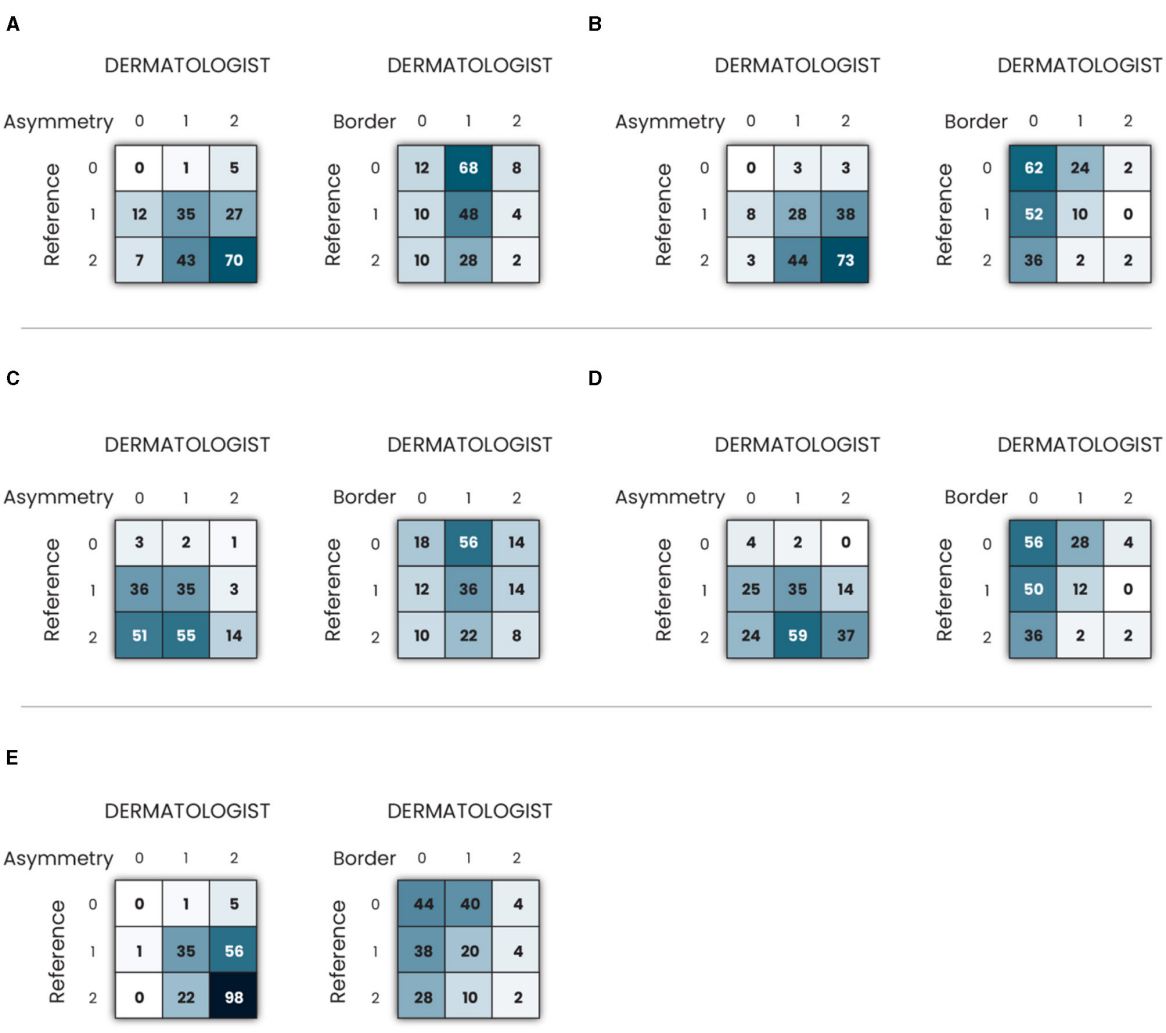
Confusion matrix of clinical criteria asymmetry and border in melanoma images using: **(A)** Grad-CAM; **(B)** Grad-CAM++; **(C)** Eigen-CAM; **(D)** Score-CAM; and **(E)** LIME.

To assess the AB clinical criteria for melanoma in our study, a confusion matrix was constructed after grading melanoma images, as depicted in Figure 5. The diagonal of the matrix signifies instances where the reference and dermatologists concurred, indicating total agreement. The off-diagonal elements, displaced either one or two columns away from the main diagonal, denote partial agreement or disagreement, respectively. The generated confusion matrix was used to construct (Table 1), presenting a comprehensive overview of the inter-rater reliability of the AB clinical criteria for melanoma in our study.

**Table 1:** Agreement between clinical ABC melanoma features and each visual explanation.

**Technique**	**Total agreement**		**Partial agreement**		**No agreement**		**Total**
**Assymetry**							
Eigen-CAM	52	26.00%	96	48.00%	52	26.00%	200
Grad-CAM	105	52.50%	83	41.50%	12	6.00%	200
Grad-CAM++	101	50.50%	93	46.50%	6	3.00%	200
LIME	115	57.50%	80	40.00%	5	2.50%	200
Score-CAM	76	38.00%	100	50.00%	24	12.00%	200
**Border**							
Eigen-CAM	62	32.63%	104	54.74%	24	12.63%	190
Grad-CAM	62	32.63%	110	57.89%	18	9.47%	190
Grad-CAM++	74	38.95%	78	41.05%	38	20.00%	190
LIME	66	34.74%	92	48.42%	32	6.84%	190
Score-CAM	70	36.84%	80	42.11%	40	21.05%	190
**Color**							
Eigen-CAM	75	37.50%	121	60.50%	4	2.00%	200
Grad-CAM	69	34.50%	123	61.50%	8	4.00%	200
Grad-CAM++	41	20.50%	132	66.00%	27	13.50%	200
LIME	29	14.50%	148	74.00%	23	11.50%	200
Score-CAM	32	16.00%	141	70.50%	27	13.50%	200
**TOTAL**							
Eigen-CAM	189	32.03%	321	54.41%	80	13.56%	590
Grad-CAM	236	40.00%	316	53.56%	38	6.44%	590
Grad-CAM++	216	36.61%	303	51.36%	71	12.03%	590
LIME	210	35.59%	320	54.24%	60	10.17%	590
Score-CAM	178	30.17%	321	54.41%	91	15.42%	590

Table 1 shows the results of total, partial, and no agreement rates to ABC melanoma rule. Asymmetry was the criterium of the highest agreement rate among the three. LIME, Grad-CAM, and Grad-CAM++ were the top techniques for asymmetry, all of them showing >50% of total agreement rates. 40–50% of all techniques showed a partial agreement rate in this criterium. Eigen-CAM had the poorest performance, with >25% of no agreement rate, while Grad-CAM ++ and LIME showed only around 3% of no agreement. Thus, Grad-CAM++ seems to be the best technique for asymmetry detection in melanoma cases.

Regarding border evaluation, all visual explanation techniques showed similar total agreement rates, between 32 and 39%, but Score-CAM and Grad-CAM++ showed no agreement in ≥20% of the cases. For partial agreement, Grad-CAM and Eigen-CAM showed the best numbers. Taking all into account, it looks like Grad-CAM is the most reliable technique to identify border abnormalities by visual maps.

As for the color match, Grad-CAM presented the top performance, with 40% of total agreement, followed by Grad-CAM++ and LIME. For partial agreement, all techniques showed similar results. As Grad-CAM had only 6% of no agreement, it was considered the best technique for this aspect.

Analyzing the three criteria together, Grad-CAM was the best visual explanation technique in agreement with the ABC rule of melanoma cases. In second and third places, respectively, are LIME and Grad-CAM++, which performed very similarly in this experiment. Eigen-CAM and Score-CAM finalized in the fourth and fifth places, respectively, Eigen-CAM presenting a little better result for total and no agreement rates.

### 3.2. Qualitative results

Comments of both dermatologists about the five different visual explanation methods can be seen in Table 2, as well as their preferable choices, and their inter-expert agreement rates. Grad-CAM and Grad-CAM++ were in the top position for both. Score-CAM was unanimous the third place in choice and the worst positions were occupied by LIME and Eigen-CAM techniques. The overall inter-expert agreement rates was 60% total and 40% partial, although they were not coincident for each explainability method. There were no disagreements.

**Table 2:** Qualitative results of each visual map technique showing the comments, ranking and inter-expert agreement.

	**Dermatologist 1**			**Dermatologist 2**		
**Visual map**	**Comments**	**Preference**	**Inter-expert**	**Comments**	**Preference**	**Inter-expert**
**technique**		**ranking**	**agreement**		**ranking**	**agreement**
Score-CAM	Poor delimitation of the lesion, very specific, but very low sensitivity	3	Total	It points only to specific areas, but not necessarily the relevant ones	3	Partial
Eigen-CAM	It creates a rectangle over the central area; does not seem specific nor sensitive	4	Total	It maps a great area, without differentiation between relevant areas; it only points to the lesion	5	Total
LIME	It creates geographical areas, hard to interpret; it can delimitate the lesion very well, but does not seem specific or sensitive	5	Total	Maps do not explain why clinically similar areas of the skin show different patterns in the map; does not seem sensitive or specific	4	Total
Grad-CAM	It delimitates the lesion most accurately, and have better match to clinically relevant areas	1	Partial	It seems more specific, but not so much sensitivity; it points correctly to the whole lesion	2	Partial
Grad-CAM++	It does not delimitate the lesion; it highlights only the major relevant areas; high specificity and low sensitivity	2	Total	It also seems more specific, localizing the relevant areas but less sensitive; it points only to parts of the lesion, not delimitating the whole area	1	Total

## 4. Discussion

Due to the difficulty of interpreting deep learning models and giving a plausible explanation for a prediction, this theme has been increasingly addressed in the literature through proposed methods, taxonomies, and benchmarks (29, 30, 55, 56). However, there is little consensus on what is interpretability/explainability in machine learning and how to evaluate it for benchmarking (55). Especially in the medical field, as physicians play a major role in endorsing (or not) the use of AI algorithms, it is important to reach out to them, understanding how and what they think about the explainability models. An adequate visual explanation should be able to identify details that help explain a particular classification (26). In this context, interpretability can be described as the degree to which a human can consistently predict the models result (25, 35).

There are very few studies addressing this question in practice. Our work is likely one of the pioneers in this field, trying to bring light to the CNN black box, through practical experiments using human experts in the field of Dermatology. Our methodology tested the discriminative visual explanation of five different techniques to support the understanding of the model's decision and our quantitative and qualitative results composed an interesting picture to compare the methods in a real-life situation.

Asymmetry was the criterium with the highest agreement rate, reaching 57.5% using LIME. This can be explained because the LIME technique is very geographical, dividing the maps lesion into several different areas and color tones, making it almost impossible to produce a symmetric visual map. As melanoma clinical lesions are often asymmetric themselves, the high agreement may be more of an expression of this fact rather than a true match with the dermatologists criterium. On the other hand, Eigen-CAM had the worst performance, justified by the fact that it often stamps a rectangle over the entire lesion, showing no asymmetry at all, poorly reflecting the reality of the clinical lesion. Grad-CAM and Grad-CAM++ also performed very well for asymmetry, with only ≤ 6% of no agreement rate and excellent numbers for high and partial agreements rate.

Borders evaluation was the criterium with the lowest agreement rate. Grad-CAM showed the best results, with only 9% of no agreement rate, followed by Eigen-CAM. That corroborates the fact that Grad-CAM was the only technique cited as better limiting the border area. Eigen-CAM might have a good result in this assessment because, as said, the rectangle displayed in the visual map included, in most cases, the border area. As described above for LIME technique in asymmetry evaluation, Eigen-CAM may not reflect a true match with the border area, but only a coincidence dependent on the techniques visual map displayed. The worst performance techniques were Score-CAM and Grad-CAM++ was showing ≥20% of no agreement rates, which was also pointed out by the dermatologists.

Color abnormalities assessment is probably the most relevant criterion when dermatologists evaluate lesions such as melanoma. Eigen-CAM and Grad-CAM presented the best results, over 30% of high agreement and ≤ 4% of no agreement. As already mentioned, Eigen-CAM, as its visual map prints a big rectangle over the lesion, it did match the color abnormalities, but indiscriminately, as pointed out by the dermatologists. Thus, for this criterium, when the qualitative study is considered, Grad-CAM seemed to better match the relevant areas of color abnormalities of the lesions. LIME and Score-CAM did poorly in this evaluation, showing only around 15% of high agreement and 12-13% of no agreement.

Overall, Grad-CAM showed the best agreement rate with 40% of total agreement and only 6% of no agreement. This was also reflected by the dermatologists opinion, which ranked it in the top two techniques. The LIME technique ended up in the second position in the quantitative study, probably because of the high performance for asymmetry, but was ranked very low by the dermatologists, in the last two spots. Grad-CAM++ turned up to be third in quantitative agreement, but it was highly ranked by the dermatologists (first and second places). Eigen-CAM performed fourth in the agreement experiment and it was disliked, as well, by the experts. Finally, Score-CAM showed the worst performance in the quantitative assessment, but it assumed a unanimous third place among the dermatologists, only after Grad-CAM and Grad-CAM++.

Another study, recently published, tested four Convolutional Neural Network models using five different interpretation techniques (saliency, guided backpropagation, integrated gradients, input gradients, and DeepLIFT) to compare their agreement with experts previous annotations of esophagus cancerous tissue, showing that saliency attributes match best with the manual experts delineations and that there was moderate to high correlation between the sensitivity of a model and the human-and-computeragreement (57).

Saliency maps are one of the few methods that can be used for visual explanations. As in our study, the evaluation of explainability with humans is ideal to assess the understanding and applicability of these methods (55). A large variety of methods have been applied for this aim. However, recent work has shown that many are, in fact, independent of the model weights and/or the class labels. In these cases, it is likely that the model architecture itself is constraining the saliency maps to look falsely meaningful: frequently, the maps just act as a variant of an edge detector. This is particularly dangerous in the context of skin cancer detection, as features at the borders of lesions are often considered diagnostic for melanoma: saliency maps that highlight the edges of a lesion may be misconstrued as clinically meaningful (51). Interestingly, our results in the experiment showed that most of the techniques fail to identify the borders of the lesions, and only Grad-CAM showed a good performance.

Although human evaluation is essential to assess interpretability, the evaluation of the human subject is not an easy task (55). In our experiment, it is not possible to measure, in a concrete way, if the techniques are looking at the same features as the experts to confirm or not the agreement. Some studies claimed that people tend to disregard information that is inconsistent with their prior beliefs. This effect is called confirmation bias (25) and that is why our dermatologists assessed the dermoscopic images and Grad-CAM visual maps separately and blindly, trying to avoid it. Also, relying only on examples to explain the models behavior can lead to over-generalization and misunderstanding (58), and observing where the network is looking at the image does not tell the user what the CNN is actually doing with that part of the image (59).

Furthermore, when evaluating the most appropriate explanation, one must take into account the social environment of the ML system and the target audience. This means that the best explanation varies depending on the domain of the application and the use case (60). Despite the fact that a saliency map located on the lesion cannot yet be viewed as justification that clinically meaningful correlations have been learned, a map that is clearly located on a clinically irrelevant region could be used to signal a prediction that should be ignored (51).

In our study, we encouraged experts to provide quantitative and qualitative analyses of the different explainability techniques to assess subjective matters related to how they visually interpreted melanoma lesions alongside the technique's results. By doing that, we touched unknown territory in terms of analyzing how useful these visual explainability techniques can be in clinical practice. In our study design, the experts gave important feedback that was statically detailed and explored. There was no adoption of a method described in the scientific literature because it was not possible to find one. In the future, it may be pertinent to carefully explore and propose study designs to address this issue, preferably exploring subjective matters objectively, minimizing model and expert biases, and focusing on the real-world gains of adopting AI algorithms in clinical practice.

## 5. Conclusion

Our work is likely one of the pioneers using experts to try to bring light to the CNN black box in the Dermatology area, performing quantitative and qualitative studies on different visual explanation techniques for melanoma. Our results demonstrated that there is a significant agreement between clinical features used by dermatologists to diagnose melanomas and visual explanation techniques, especially Grad-Cam. The interpretation of black-box generalization in melanoma images based on visual maps showed up to be promising, presenting trustworthy outputs compared to experts interpretations and encouraging new studies.

## Data availability statement

The raw data supporting the conclusions of this article will be made available by the authors, without undue reservation.

## Ethics statement

The studies involving humans were approved by Hospital Israelita Albert Einstein. The studies were conducted in accordance with the local legislation and institutional requirements. The participants provided their written informed consent to participate in this study.

## Author contributions

MG-B, WV, and VF had the idea, designed the experiments, wrote, and reviewed the final manuscript. MG-B and AO performed the experiments and reviewed the final manuscript. RS reviewed the literature and developed the CNN. BM overviewed the entire process, was responsible for accessing the funding, and reviewed the final manuscript.
